# A Game Theory-Based Obstacle Avoidance Routing Protocol for Wireless Sensor Networks

**DOI:** 10.3390/s111009327

**Published:** 2011-09-29

**Authors:** Xin Guan, Huayang Wu, Shujun Bi

**Affiliations:** 1 School of Information Science and Technology, Heilongjiang University, Harbin 150080, China; E-Mails: wuhy_2000@yahoo.com.cn (H.W.); bishujun@163.com (S.B.); 2 Department of Information and Computer Science, Keio University, Yokohama 223-8522, Japan

**Keywords:** wireless sensor networks, obstacle avoidance, game theory, Nash equilibrium

## Abstract

The obstacle avoidance problem in geographic forwarding is an important issue for location-based routing in wireless sensor networks. The presence of an obstacle leads to several geographic routing problems such as excessive energy consumption and data congestion. Obstacles are hard to avoid in realistic environments. To bypass obstacles, most routing protocols tend to forward packets along the obstacle boundaries. This leads to a situation where the nodes at the boundaries exhaust their energy rapidly and the obstacle area is diffused. In this paper, we introduce a novel routing algorithm to solve the obstacle problem in wireless sensor networks based on a game-theory model. Our algorithm forms a concave region that cannot forward packets to achieve the aim of improving the transmission success rate and decreasing packet transmission delays. We consider the residual energy, out-degree and forwarding angle to determine the forwarding probability and payoff function of forwarding candidates. This achieves the aim of load balance and reduces network energy consumption. Simulation results show that based on the average delivery delay, energy consumption and packet delivery ratio performances our protocol is superior to other traditional schemes.

## 1. Introduction

Wireless sensor networks (WSNs) are composed of many tiny devices, each with sensing and data storing, processing and communication capabilities. Sensor networks have many potential applications, such as battlefield surveillance, environmental monitoring and industrial asset management. However, the design of energy efficient, scalable routing algorithms remains a challenging issue for researchers.

Recently, some research efforts have focused on establishing efficient routing paths for transmitting packets from a sensor node to a sink in wireless sensor networks [1]. Shortening the route length can help reduce the transmission overhead and delay time, as well as increase the packet delivery ratio. However, the existence of obstacles may either impede the establishment of a routing path or increase the route length. Generally, any geographic region where sensing and communication are not possible can be treated as an obstacle. In wireless sensor networks, obstacles can be formed dynamically owing to several reasons [2]: firstly, the random deployment of sensors can causes a non-uniform distribution of wireless sensor networks, where some regions might not contain any sensor nodes. Secondly, some sensor nodes exhaust their power after working for a relatively long period of time, resulting in regions without any sensing and communication functionality. Thirdly, physical obstacles such as mountains or buildings may also be encountered. Fourthly, sensor nodes in some regions may not work normally owing to malicious signal interference. Finally, passing groups of animals or a strong breeze blowing on the sensor networks can also cause some sensor nodes to fail or be swept away, dynamically forming an obstacle. Once obstacles appear, packet routing will be blocked or become inefficient. In the absence of any appropriate obstacle avoidance routing technique, this would lead to a situation where packets may get lost in the network, wasting node energy and disabling communications between some node pairs.

Existing obstacle handling techniques mainly use the following two approaches to solve the problem: the right hand rule [3] and the backpressure rule [4]. Some recent protocols are also based on the right hand rule-bypass packets to the sink approach [5,6]. According to the right hand rule, packets tend to be forwarded along the boundaries of obstacles. The probability that the obstacle boundary nodes are then shared by several communication sessions is higher than for other nodes. This results in excessive energy consumption and data collisions within these nodes. In [7], an anchor node is proposed to solve the routing problem with obstacles in sensor networks. This approach can mitigate excessive energy consumption and reduce the collision of boundary nodes, since packets would be forwarded through anchor-based virtual circles. However, the obstacle diffusion issue still occurs in the network initialization phase.

In this paper, we propose a novel protocol to handle the obstacle problem based on forwarding mechanisms in sensor networks. Our protocol first computes the connectivity degree set for every node and separates the degree set into out-degree and in-degree sets. Given that some nodes may have zero out-degree, we establish a concave region in the sensor network. Packets cannot be forwarded to the concave region and this approach can avoid lost packets and reduce the transmission delay. Secondly, during the phase of forwarding node selection of source nodes, the source node considers residual energy, out-degree and forwarding angle of forwarding candidates and infers the forwarding probability according to these three factors. Furthermore, we provide a game theory model based on the forwarding probability of forwarding participants and prove that in the game model a Nash equilibrium exists.

The rest of this paper is organized as follows: Section 2 presents related works on obstacle based routing and game theory-based protocols in wireless sensor networks. Section 3 presents the network initialization and how to form the concave region. In Section 4, we present the game theory model and prove that a Nash equilibrium exists in the game model introduced. Finally, we perform simulations to evaluate the performance and conclude this paper in Sections 5 and 6.

## 2. Related Works

To the best of our knowledge, this paper is the first to present the obstacle avoidance routing issue for wireless sensor networks from a game-theory perspective. Therefore, we summarize the obstacle avoidance routing and game theory based routing literature.

### 2.1. Networks with Obstacle Routing

Obstacle-avoidance routing algorithms in wireless sensor networks have been widely studied [8]. However, many of these routing algorithms are derived from Internet routing mechanisms and they do not work well in large scale sensor networks. Geography-based routing algorithms [9–11] have been investigated for providing low-complexity routing algorithms that are scalable and stable. In these schemes, packets are routed to the location of the destination node by a greedy algorithm. However, if there are obstacles in such kinds of schemes, greedy or shortest path routing would lead to a “local minimum”. For such situations, some researchers have introduced techniques such as planarization and face-traversal to forward packets around the obstacles. Meanwhile, the authors of [12] have demonstrated that traditional shortest path algorithms and greedy geographic algorithms can lead to heavy throughput losses, especially at the borders of obstacles. During the routing discovery process, source-destination links set up routes without knowledge of other flows in the sensor networks, so greedy or shortest path routing can lead to spatial congestion. Some strategies to forward around obstacles were introduced in [13]. However, such schemes are likely to fail in networks with typical obstacle configurations, and even when they work, they may provide lower throughput.

Geometric obstacle avoidance is proposed in [14]. It uses the geometric properties of a node to determine if a message can be stuck at that node. An algorithm is developed to find holes in the network, defined as areas of the network bounded by the stuck nodes. The disadvantage of this technique is the high complexity of hole detection. Additionally, this does not guarantee delivery when the destination is inside the hole.

The cost based approach [15] consists in assigning a cost to each node, proportional to the distance to the destination. When greedy forwarding fails, a node will forward a packet to a neighbor with a lower cost than itself. Although the complexity and the overhead of the algorithm is average, it does not choose optimal paths. Flooding based techniques [15,16] use broadcasting to forward the message once a packet is stuck. Although their complexity is low, the overhead is high. Although they guarantee delivery, path optimality is not a concern.

Hybrid techniques use at least two obstacle avoidance techniques. The motivation is the improved efficiency of the path and the guaranteed delivery of the message, and they are used when only one of the two techniques is not enough to achieve these requirements. The disadvantage is the increased overall complexity. In [17], a protocol combining greedy routing and adaptation of the transmission range to bypass obstacles is described. Indeed, the protocol manages to “jump over” obstacles, but the routing path created is not optimal and the energy costs can become high, In [18], the authors propose a variation of the right hand rule and manage to bypass even hard obstacles. The paths created are quite efficient, but not optimal.

### 2.2. Networks with Game Theory Based Routing

Game theory has been used in the past as a model to study different aspects of computer and communication networks. Recently, more and more researchers have focused on developing wireless network algorithms using game theory. The most important application of game theory to wireless sensor networks is routing algorithm design. In [19], the authors used game theory to analyze the outcome of a game in which the deployed sensors belong to different sinks and can receive incentives for cooperative forwarding. The authors in [20] proposed a reliable query routing scheme. In their approach, the candidate nodes are modeled as rational nodes cooperating to discover the optimal network architectures that can maximize the payoff in a game. Here, node payoff means the benefit of a node's action minus its costs. In [21], the authors consider the issue of packet forwarding in multi-class sensor networks using game theory.

In the incentive mechanism of wireless networks routing protocols, *Ad-Hoc* VCG [22] is a fundamental and classical method. This algorithm introduced the economic theory and adopted the classical VCG auction model for wireless *ad-hoc* networks. The main target of *Ad-Hoc* VCG is how to incite the intermediate nodes to bid their real cost for forwarding packets. *Ad-Hoc* VCG is based on monetary transfer and has several nice features: it discovers the most energy-efficient path between the source and the destination, and it is truthful and it stimulates the nodes to behave according to the protocol specification. The protocol proposed by the authors of [23] considers both route discovery and packet forwarding on the computed routes. The authors introduce a novel solution concept called cooperation-optimal protocol and prove that it is optimal for a selfish user to fulfill the routing decision in the packet forwarding phase. In [24], the authors introduce a Nash equilibrium-based mechanism named OURS. OURS gives a perfect solution for excessive payoff issues under the VCG mechanism. In the VCG model, the algorithm achieves strategy-proofness for any nodes. In OURS, the protocol releases the restriction of strategy-proofness, and proves the existence of a Nash equilibrium.

## 3. Network Initialization

**Figure 1.**
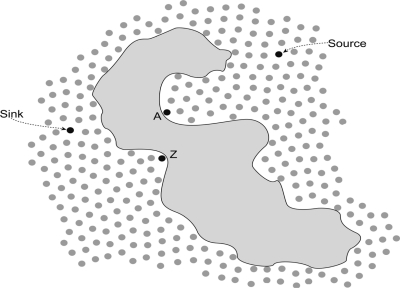
Illustration for the node out-degree is zero and in-degree is zero cases in sensor networks with obstacles.

In our work, since the nodes are static, all nodes know their own locations before network initialization. In the initialization stage of the network, each node sends its own location information to its one hop neighbors. Meanwhile, each node also receives all the location information from all its one hop neighbors. When nodes acquire their neighbor location information, they compute the distance between themselves and the sink, and the distance between every one hop neighbor and the sink. Then, each node compares those distances and concludes which node is closer than itself to the sink, then it counts the number of one hop neighbors closer to the sink in Euclidean distance between nodes, and this number is its out-degree number. These nodes are then included in the out-degree set. The rest of the nodes that are not in the out-degree set constitute the in-degree set. Obviously, nodes in the in-degree set are farther away from the sink. After the computation and comparison are finished, each node has acquired out-degree and in-degree sets of their own. There are two extreme cases that could happen for the out-degree set or the in-degree sets of some nodes. Figure 1 shows the illustration of this for the cases where the out-degree of a node is zero and in-degree of a node is zero.

1. The in-degree of some node is zero. From Figure 1, we can see that *Z* is a node whose in-degree is zero and it is located at the boundary of the obstacle convexity. We can conclude that this node has no forwarding task. Therefore, in this paper, we do not take into consideration such kinds of nodes.
2. The out-degree of some node is zero. In Figure 1, *A* is a node whose out-degree is zero. This kind of nodes is extremely special and their number is exiguous, but we cannot neglect the existence of such nodes.

**Figure 2.**
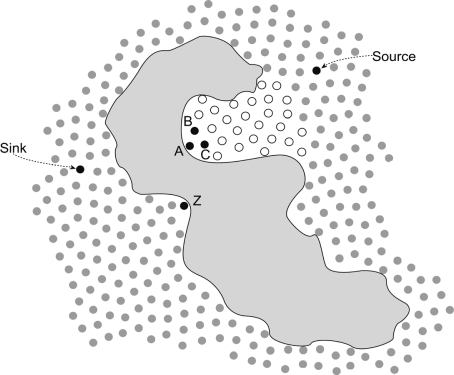
Construction of a forbidden region for a concave region.

The existence of a zero out-degree node can have a fatal influence on the routing of wireless sensor networks. Thus, we present a separate discussion of these nodes. From Figure 1, we can see that nodes whose out-degree is zero are always located at the extreme points of the concave boundary of obstacles. When some nodes discover their out-degree is zero, they send a packet to all neighbors within one hop and the packet includes its location, unique *ID* and connectivity (out-degree and in-degree). Once the neighbor nodes receive the packet, they would prune off one degree from their own out-degree set. Figure 2 shows the concave region when an obstacle exists in a sensor field. From Figure 2, we can see that the node *A* has two neighbors (node *B* and *C*) that have only one forwarding link to node *A*. If they receive a packet from *A*, they prune off one degree from their out-degree set. Meanwhile, their out-degree sets would also change to zero. Nodes *B* and *C* would reduplicate the operation that node *A* did once: send a packet to its neighbor, and so on, we would obtain a concave region from the composition of the nodes whose out-degree is zero. In Figure 2, the region formed by white dots (out-degree is zero) is the concave region. Based on the out-degree, we can divide the nodes into two categories, zero out-degree nodes and non-zero out-degree nodes.

## 4. Game Model

In this paper, when a source node *S* wants to transmit a packet to the destination *D*, the nodes included in the out-degree set of *S* are the candidate nodes that could be the forwarding node. Node *S* would transmit packets to all the nodes included in its own out-degree set. These candidate nodes would make decision of participating in forwarding or remaining silent based on their forwarding probabilities and the payoff function.

### 4.1. Forwarding Probability

**Figure 3.**
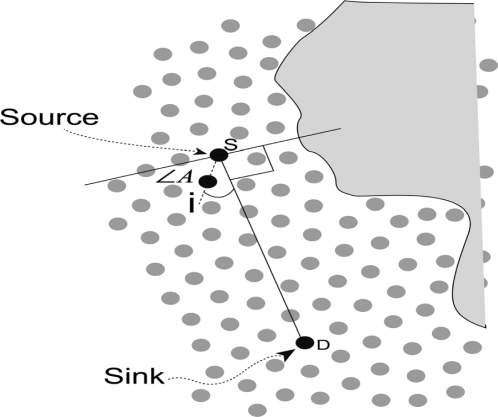
Illustration for forwarding angle with source, destination and forwarding candidate node.

Forwarding candidate nodes would take the following three factors into consideration to form their forwarding probabilities:

1. The ratio composed by the out-degree of their own and the average out-degree for all nodes. We denote it as *O_i_/O_a_*. Here, *O_i_* means the out-degree of node *i* and *O_a_* means the average out-degree for all nodes in the sensor field.
2. The ratio composed by current energy and initial energy (*E_{current}_/E_{initial}_*) of nodes. We denote it as *E_i(ci)_*.
3. The cosine value of the angle composed by the line linked candidate node *i* and source *S* and the line constituted by source node *S* and destination *D*. It is shown in Figure 3 and we denote it as cos *A*.

For any candidate node *i*, it it easy to obtain *E_i(ci)_*. From Figure 3, we can deduce the cosine value of the angle between line *iS* and *SD* by Equation (1). Since we assume that the nodes are discrete and uniformly distributed, it is easy to obtain *O_i_/O_a_* for any node:

(1) $$\cos\;A=({\bar{\mathrm{iS}}}^{2}+{\bar{\mathrm{iD}}}^{2}-{\bar{\mathrm{SD}}}^{2})/2*\bar{\mathrm{iS}}*\bar{\mathrm{iD}}$$

*Lemma 1*: The angle composed by the line constituted by candidate node *i* and source *S* and the line constituted by source node *S* and destination *D* is less than 90 degrees.

*Proof*: In Figure 3, we can consider the node S as the source and construct a line from *S* to *D*. We assume that there is a candidate node of *S* and we denote it as *i*. In case the angle composed by the line constituted by candidate node *(i)* and source *(S)* and the line constituted by source node *S* and destination *D* is more than 90 degrees, the distance between *i* and *D* (*iD*) must be more than the distance between *S* and *D* (*SD*). In case *iD* > *SD* and here, *iD* is the distance from node *i* to destination *D*, *SD* is the distance from source node *S* to destination *D*, *i* must be the in-degree node of *S* and has no qualification to be the candidate node to participate in forwarding. This contradicts the supposition, and the Lemma is proven.

∠*A* is the angle between the candidate node and the line *SD*. We have concluded that the angle between the candidate node and the line *SD* is at the degree interval [0, 90] from Lemma 1. For the cosine value of ∠*A*, the cosine value is a decreasing function in the interval [0, 90]. In other words, ∠*A* = 0°, cos *A* = 1; ∠*A* → 90°, cos *A* → 0.

*Lemma 2*: In terms of load balance and energy consumption, minimum angle routing has better performance than shortest path routing.

**Figure 4.**
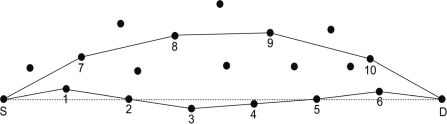
Illustration for different routing path according to minimum angle and shortest path.

*Proof*: In Figure 4, *S* is the source and *D* is the destination node. Line *SD* is the linear distance between *S* and *D*. *A_SD_* is the forwarding path selected by the minimum angle routing and *G_SD_* is the forwarding path selected by the shortest path routing. *A_SD_* = 1 → 2 → 3 → 4 → 5 → 6; *G_SD_* = 7 → 8 → 9 → 10. According to the radio model [25], we can calculate the energy consumption of link *A_SD_* and *G_SD_*. It is shown by Equations (2) and (3):

(2) $$A_{\mathrm{SD}}\;=\;14{\mathrm{KE}}_{\mathrm{elec}}+{Kɛ}_{\mathrm{fs}}d_{S\to 1}^{2}+K{ɛ}_{\mathrm{fs}}d_{6\to D}^{2}+{Kɛ}_{\mathrm{fs}}\sum_{i=1}^{5}d_{i\to i+1}$$

(3) $$G_{\mathrm{SD}}\;=\;10{\mathrm{KE}}_{\mathrm{elec}}+{Kɛ}_{\mathrm{fs}}d_{S\to 7}^{2}+{Kɛ}_{\mathrm{fs}}d_{10\to D}^{2}+{Kɛ}_{\mathrm{fs}}\sum_{i=7}^{10}d_{i\to i+1}$$

In the two equations above, *K* represents the packet size for transmission. *E_elec_* denotes the energy consumption of the wireless circuit for sending and receiving packets. The energy consumption of the amplifier is denoted by *ɛ_fs_*, which is decided by the distance from the sender to the receiver as well as the acceptable bit error rate. *d_i_* → *j* denotes the distance between node *i* and node *j*. For the energy consumption of nodes in sensor networks, long distance packet transmission accounts for the major portion of the energy consumption. From Figure 4 we can see that, although the link *A_SD_* passes through more nodes than link *G_SD_*, in the aspect of transmission distance, link *A_SD_* is shorter than link *G_SD_*, therefore the energy consumption for *A_SD_* is less than for *G_SD_*. Furthermore, six nodes share the total energy consumption of *A_SD_*, and for *G_SD_*, only four nodes participate in the forwarding. From a load balance point of view, every node located in *A_SD_* would cost less energy than those located in *G_SD_*. Thus, our algorithm achieves the goal of load balance for sensor networks. The average energy consumptions for each node separately located at link *A_SD_* and *G_SD_* are *A_SD_*/6 and *G_SD_*/4. Based on the above description, we can conclude that the energy consumption of link *A_SD_* is less than that of link *G_SD_*. The average energy consumption for each node located at link *A_SD_* is less than that of link *G_SD_*.

For any node *i*, we consider Equation (4) as the forwarding probability and *α* is a variable parameter, the interval of *α* is (0, 1). In order to choose the most effective and energy efficient routing path to finish the forwarding task, we combine the two important issues under consideration: Energy residual and minimum angles. As for Equation (4), we proposed the weight value function *P(i)* to calculate the forwarding probability for each candidate. As for the parameter *α*, we only assume it should be within the interval (0, 1), and it could work in our weight value function:

(4) $$P(i)=\left[\alpha *E_{i(ci)}+(1-\alpha )*\cos\;A\right]*(O_{i}/O_{a})$$

### 4.2. Payoff Function

We define that for each forwarding process in one game, the forwarding participants of the source node are in the out-degree set. The strategy space of participants is *S_i_* = {0, 1}, where pure strategy “0” means that node *i* would not forward a packet and “1” means to forward the packet. In our algorithm, node *i* possesses the mixed strategy based on pure strategy space *S_i_*. It means that node *i* would forward packets according to the probability *P(i)*.

During the game progress, different strategies lead to different benefits for each participant. The benefit to participants is described by the payoff function. For any participant, the benefit has some relation with strategy. We assume the strategy combination is *s* = (*P*(*i*), *P*(*−i*)) in one game, where *P*(*i*) means the strategy of participant *i* and also the forwarding probability of nodes, and *P*(*−i*) = *P* (*1*), …, *P*(*i − 1*), *P*(*i + 1*), …, *P*(*n*) means the strategy of other participants. We denote the payoff of *i* in this strategy combination by *u_i_*(*P*(*i*), *P*(*−i*)). Here, the payoff for any participants is the expected benefit with a certain participation probability. It is composed of the benefit *B_i_* (*P*(*i*), *P*(*−i*)) and the cost *C_i_*(*P*(*i*), *P*(*−i*)) of *i*. Theoretically, only the nodes that participate in forwarding would cost and gain the benefit and for the nodes that do not forward packets, they cannot gain any benefit and the payoff is also zero. We define the payoff function of participant *i* by Equation (5):

(5) $$u_{i}(P(i),P(-i))=\{\begin{array}{cc} \frac{B_{i}(P(i),P(-i))}{C_{i}(P(i),P(-i))}, & \text{forwarding}\\ 0, & \text{otherwise} \end{array}$$

In the game period, each participant would like to use its optimal strategy to maximize its payoff. Therefore, the benefit and cost of the participant would impact the strategy for all the nodes located on the routing path. Furthermore, it would impact the transmission reliability grade of the routing path. We consider the transmission reliability grade as the benefit of intermediate nodes. We assume for strategy combination *s*, in case the next hop node is *j* for intermediate node *i*, the benefit of *i* is shown by Equation (6):

(6) $$B_{i}(P(i),P(-i))=R_{\mathrm{up}}*R_{\mathrm{ij}}*R_{j}$$

where *R_up_* is the transmission reliability grade of source to destination, *R_ij_* is the transmission reliability grade between node *i* and *j*, *R_j_* is the transmission reliability grade from neighbor *j* to the sink. We consider the energy consumption of intermediate noded and the shortening of network lifetime as the cost for forwarding participants. Apparently, *C_i_*(*P*(*i*), *P*(*−i*)) would be augmented according to the reduction of network consumption and the prolonging of network lifetime. In the strategy of *i* participating in forwarding, residual energy of *i* and *j* are the evaluation factor for network lifetime. Therefore, we define the cost of *i* for strategy combination *s*. This is shown by Equation (7):

(7) $$C_{i}(P(i),P(-i))=\frac{(E_{i}^{\mathrm{rx}}+E_{i}^{\mathrm{tx}})*F_{i}}{L*P_{i}+L*P_{j}}$$

where $E_{i}^{\mathrm{rx}}$ is the receiving energy consumption of *i*, $E_{i}^{\mathrm{tx}}$ is the transmission energy consumption of *i*, *F_i_* is the intermediate packet capacity of *i*, *L × P_i_* and *L × P_j_* are the residual energy of *i* and *j*, respectively. Based on Equations (5–7), we have a combination optimization transmission reliability grade. The payoff function of forwarding participants is given by Equation (8):

(8) $$u_{i}(P(i),P(-i))=\{\begin{array}{cc} \frac{R_{\mathrm{up}}*R_{\mathrm{ij}}*R_{j}*(L*P_{i}+L*P_{j})}{(E_{i}^{\mathrm{rx}}+E_{i}^{\mathrm{tx}})*F_{i}}, & \text{forward}\\ 0, & \text{otherwise} \end{array}$$

### 4.3. Nash Equilibrium Analysis

*Theorem 1*: For a dynamic routing game *G*, there are *n* nodes participating in the game. Then in *G* there exists a pure strategy Nash equilibrium.

*Proof*: For a dynamic routing game, since the number of participants n is finite, and no matter how large the communication radius of a node is, the number of neighbor nodes is finite within the communication range. Therefore, for the pure strategy space routing game, the strategy of a participant is finite and *G* is a finite game. Since in *G*, we think that every node participating in a current game knows the strategy of previous participating nodes, *G* is a perfect information game. Furthermore, we can conclude that there exists at least one pure finite Nash equilibrium in *G* and *G* is a finite perfect information game. Besides the existence of a Nash equilibrium, we conclude that the relationship among combination optimization transmission reliability grade, energy consumption and network lifetime in a routing game.

*Theorem 2*: For a dynamic routing game *G* that has n nodes participating, the optimal path P* regarding energy consumption and network lifetime as aims is a Nash equilibrium result of *G*.

*Proof*: According to the description of payoff function, in the routing game the benefit of every participant gained has a relationship between combination optimization transmission reliability grade, energy consumption and network lifetime. It means that the higher the optimization, the larger the payoff value for a participant. Apparently, the path P* is the optimal one for all paths. In path P*, the nodes have the largest payoff. Here for all the nodes located at P*, their strategies are optimum. In the case that the strategy of other participants does not change, even if some node located at P* changes its strategy, the payoff would not augment. It means that the participants located at P* have no appetite to change their strategies. For the nodes not located at P*, they do not participate in forwarding packet, thus their payoff is zero. In this case, for all the participants, if they do not change their strategies, the participants not located at P* cannot join the set of intermediate nodes even though they change their strategy. This is because the node located at P* would not select the node not located at P* as next hop. Therefore, the nodes not located at P* have no appetite to change their strategies. Based on the above description, in the routing game, there are no participants having appetite to change their strategies. Therefore, P* is a Nash equilibrium result of *G*.

## 5. Performance Evaluations

In this section, we make a simulation to evaluate the performance of obstacle modeling. Firstly, we introduce the performance metrics and the simulation scenarios. Then we evaluate the system performance with the given scenarios and parameters. Finally, we show the performance comparisons between our algorithm and SPEED [4] and GPSR [3].

### 5.1. Simulation Metrics and Scenarios

We use three metrics to evaluate the performance. The average delivery delay, energy consumption and packet delivery ratio. The size of the sensor network is set to 200 m × 200 m where 100 nodes are uniformly distributed. An obstacle is created at the center of the area; *i.e*., an area is set with no nodes inside to simulate an obstacle in a realistic environment. We select IEEE 802.11 as our MAC protocol and the radio range of nodes is 40 m. The sink is located at the left side of the obstacle. We give two comparison sets to show the performance. The first one is based on the transmission rate of the source node is gradually increased until it reaches 100 packets per second. The second one is based on an expanding obstacle area. During the process of simulation, there is always some node that gains a forwarding opportunity in one game. In other words, some node would be the forwarding node. Therefore, after the destination received the packet from the source node, each forwarding node would change their own benefit according to the payoff function.

### 5.2. Simulation Results

#### 5.2.1. Comparison on Increasing Transmission Rate

**Figure 5.**
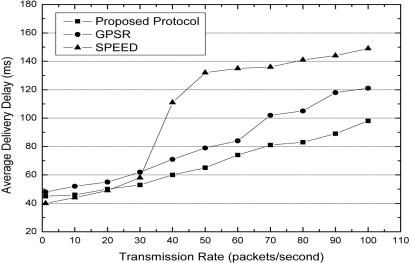
Average delivery delay with different transmission rates.

Figure 5 shows the average delivery delay with increasing transmission rate. The average delivery delay means the average time delay between the moment the source sends a packet and moment the destination receives this packet. When the transmission rate is 1 packet per second, we can see that the average delivery delay of SPEED is lower than our protocol and GPSR. This is because SPEED always tries to discover a high speed path for forwarding packets. Since the transmission rate increases, the average delivery delay of SPEED increases significantly. This is because congestions occur at the boundary in SPEED. In our protocol, when the packets need to bypass the obstacle, the boundary candidate nodes have a lower forwarding probability than normal nodes. This is because the size of out-degree of boundary nodes is less than that of the out-degree of normal nodes. In the forwarding node selection game, the probability that a great amount of packets are forwarded by the same node is relatively low. Thus, the average delivery delay of our protocol does not significantly increase with an increase in transmission rate.

**Figure 6.**
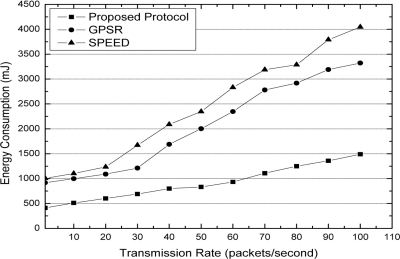
Energy consumption with different transmission rates.

Figure 6 shows the energy consumption of the three protocols. For GPSR and SPEED, the source always selects the node closest to the destination in the neighbor set. However, normally the closest node is the local superior decision, not the global optimal decision. This has been proven by lemma 2. For our protocol, in the forwarding node selection game, if some node has a lesser angle with the line formed by source and destination, it has the high probability to be the forwarding node. Thus, our protocol costs less node energy.

**Figure 7.**
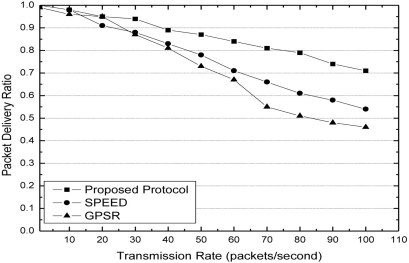
Packet delivery ratio with different transmission rates.

At the aspect of packet delivery ratio, from Figure 7 we can see that our protocol has better performance than SPEED and GPSR. With the increase of transmission rate, SPEED and GPSR always forward packets along the obstacle boundaries by perimeter approach. This leads to a high probability of packet congestion around the obstacle. In our protocol, since the process of forwarding node selection is a game process, the source has lower probability to make the same candidate gain too much benefit from the game process. This is the reason why the packet delivery ratio of our protocol does not significantly decrease with the increase of transmission rate.

#### 5.2.2. Comparison in an Expanding Obstacles Scenario

In this section, we compare SPEED, GPSR and our algorithm under different obstacle area conditions on the following aspects: average delivery delay, energy consumption and packet delivery ratio. There are 100 nodes in the sensor field and the transmission rate is 50 packets per second. The initial area of the obstacle for the whole sensor field is 5% and it finally increases to 50% and the shape of the obstacle does not change. For the instance where the obstacle is expanding, the transmission rate and the number of nodes are constant. Moreover, the area of the sensor field is constant. It means that, the density of nodes is becoming higher per unit time. Therefore, we only need expand the area of the obstacle in the sensing field, we can compare two aspects of performance: expanding obstacle and higher density. Concerning SPEED, GPSR and our algorithm, when the area of the obstacle is changed, the performance of delivery delay, energy consumption on the boundary and the packet delivery ratio would have a significant influence. In the following, we will introduce the performance comparison with changing obstacle area.

**Figure 8.**
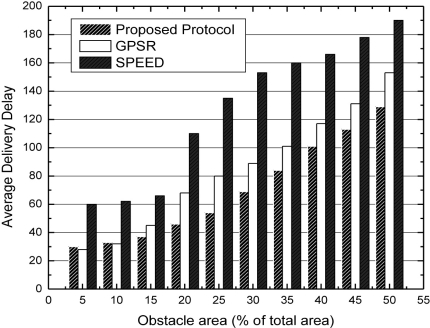
Average delivery delay with expanding obstacle area.

In Figure 8, the X axis is the changing range of obstacle area and the Y axis is the average delivery delay. In the initial phase, the obstacle area is only 5% of the whole sensor field. We can see that because of the transmission rate, the average delivery delay of SPEED is obviously becoming higher. Concerning GPSR and our algorithm, the average delivery delay is relatively low. The reason why SPEED has a higher delay is that once the congestion occurs in some path, SPEED would adopt the backpressure mechanism to balance the congestion to other paths. Therefore, it leads to the result of higher delivery delay for packets. With the expansion of the obstacle area, the influence becomes more obvious, and the density is also becoming higher. From Figure 8, we can see that the performance of average delivery delay of our algorithm is better than SPEED and GPSR. Because the obstacle is expanding, the probability of passing the zero out-degree nodes is becoming higher for the packets. Based on this condition, SPEED and GPSR need more intermediate forwarding of their packets to depart from the concave region. However, concerning our algorithm, this problem had been solved in the network initialization phase.

**Figure 9.**
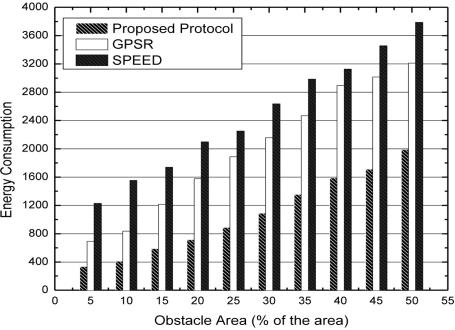
Energy consumption with expanding obstacle area.

In Figure 9, we compare the energy consumption of boundary nodes in SPEED, GPSR and our algorithm under the expanding obstacle condition. From Figure 9, we can see that when the obstacle is expanding, the nodes expend more and more energy. The main reason is that more and more nodes are located at the boundary of the obstacle. In our algorithm, we try to avoid packet forwarding by the boundary nodes and achieve the goal of load balance. Therefore, the performance of energy consumption is better than that of SPEED and GPSR.

**Figure 10.**
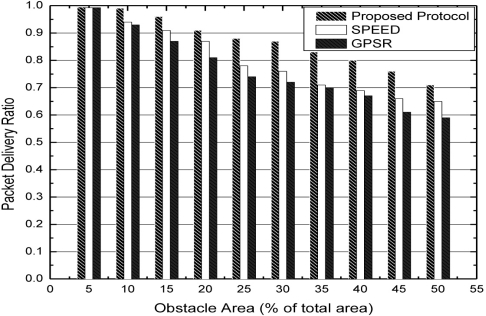
Packet delivery ratio with expanding obstacle area.

We next compare algorithms under the condition of expanding obstacle with packet delivery ratio in Figure 10. Concerning our algorithm, the probability of congestion occurring is lower because the game process and the packet delivery ratio is relatively higher. Concerning GPSR, with the expanded obstacle, the concave region is also expanding. Then, the probability of packets entering the concave region is higher and these packets need to be forwarded by the boundary nodes. This would lead to the result that the packet delivery ratio would become lower. Concerning SPEED, although it also adopts a greedy algorithm like GPSR, once the congestion occurs, SPEED would inform the upstream nodes to set up a new path for forwarding packets. This could relatively improve the performance to some extent. From Figure 10, we can see that our algorithm has better performance on packet delivery ratio than SPEED or GPSR.

## 6. Conclusions and Future Work

In this paper, we introduce a game theory-based obstacle avoidance scheme. In the process of network initialization, we use the connectivity property of nodes to determine the concave region that cannot forward any packets. This approach improves the transmission success rate and decreases the transmission delays of packets. In the aspect of setting up the routing path, we consider the residual energy, out-degree and forwarding angle of forwarding candidates. We conclude the forwarding probability and payoff function of forwarding participants. Finally, we prove the existence of s Nash equilibrium for the proposed game model. In our future, we plan to implement our algorithm in s real application scenario to verify the effectiveness in the real world. Also, in this paper, we assume that all nodes are stationary. There are some application scenarios where we need the nodes to be able to move. In such a case, we will need to consider the nodes’ mobility in our future work.
